# Perils of Investigation

**Published:** 1893-01

**Authors:** 


### PERILS OF INVESTIGATION.

Rev. Minot J. Savage, in writing of his investigations of the occult forces, truly says :—

If one wants peace in this world, the "safe" thing is to stick to the old, reputable ways endorsed by the majority "common sense" of the place where he happens to live. Only, if everybody had always done that, humanity would never have got out of the jungles or into clothes. So, fortunately for mankind, there is always some restless fellow, like the character in Dickens, who "wants to know." He is willing to defy the "common sense" of the hour for the sake of trying to get his questions answered. But this same common sense of the hour is not going to be outraged unavenged. The man who dares to know more than the average has to pay for his temerity. And he will be very fortunate indeed if he do not have to pay toll (of heart-ache, loneliness and reputation) in more directions than one.

The wide field of thought thus opened is too large to be traversed in one newspaper article. But I have a special reason for wishing to say a few things as to the perils that beset investigation in the psychical field.

1st. That one must dare the disapprobation of his "religious" friends who hold that all things that it is proper for any body to know are already "revealed," and that if there are any "spirits" they are sure to be evil ones,—all this goes without saying.

2d. Then there are the square-toed materialists who will have their shy at you. If the universe is purely a piece of mechanism and as well regulated as a machine that has run so long might be presumed to be, is it not a little curious that out of this machine should have come so many supernormal fancies to disturb the orderly people who assume to have it in charge?

3d. Then there are the friends who privately think you are a fool to want any more proof of immortality than the personal "consciousness" which they claim to possess that they are immortal.

4th. Then, again, there are the ones who, on the basis of one unsuccessful sitting and a few newspaper "exposures," have come to "know" that the whole business is a humbug. The investigator must be content to have these people look down upon him with a sort of pitying condescension.

5th. But there is one other thing that is harder to bear than either of these. And it is concerning this that I wish to free my mind a little through the columns of your paper.

I have never had any esoteric doctrines that I have supposed the world was not ready for. A noted clergyman once said to me: "What I think in my study is one thing, and what I think it is wise and best to give the people on Sunday is another thing;" and I have felt a contempt for this particular man ever since. Who am I that I should assume to be so wise that the Almighty has taken me into his confidence and trusted me with secrets that the world is not "ready for?" Poor world! That it should need to be fed on lies so long because of the weakness of its digestion! Poor God! That he should make so many things true that it is not safe for people to know! I fear I am a poor person to entrust with this sort of secrets. If the Lord doesn't want me to tell anything that is true, he had better not let me find it out! It seems to me such a pitiful insult to God to suppose he has made a lot of things true that, at the same time, are not safe.

